# Cullin-3 and Regulatory Biomolecules Profiling in Vitiligo: Integrated Docking, Clinical, and In Silico Insights

**DOI:** 10.3390/biom15071053

**Published:** 2025-07-21

**Authors:** Hidi A. A. Abdellatif, Mohamed Azab, Eman Hassan El-Sayed, Rwan M. M. M. Halim, Ahmad J. Milebary, Dhaifallah A. Alenizi, Manal S. Fawzy, Noha M. Abd El-Fadeal

**Affiliations:** 1 Medical Biochemistry and Molecular Biology Department, Faculty of Medicine, Suez Canal University, Ismailia 41522, Egypt; noha_abdelfadeal@med.suez.edu.eg; 2 Oncology Diagnostic Unit, Faculty of Medicine, Suez Canal University, Ismailia 41522, Egypt; 3 Medical Biochemistry and Molecular Biology Department, Faculty of Medicine, King Salman International University, Tur Sinai 46618, Egypt; 4 Dermatology, Venereology and Andrology Department, Faculty of Medicine, Suez Canal University, Ismailia 41522, Egypt; mohamed.azab@med.suez.edu.eg; 5 Clinical Pathology Department, Faculty of Medicine, Suez Canal University, Ismailia 41522, Egypt; eman.hassan@med.suez.edu.eg; 6 Faculty of Medicine, Alexandria University, Alexandria 21526, Egypt; rwan.mostafa1383@alexmed.edu.eg; 7 Department of Medical Laboratory, King Fahad Armed Forces Hospital, Jeddah 23311, Saudi Arabia; amilebary@kfafh.med.sa; 8 Department of Medicine, Faculty of Medicine, Northern Border University, Arar 91431, Saudi Arabia; daalenizi@nbu.edu.sa; 9 Center for Health Research, Northern Border University, Arar 73213, Saudi Arabia; 10 Biochemistry Department, Ibn Sina National College for Medical Studies, Jeddah 22421, Saudi Arabia

**Keywords:** cullin-3 (CUL3), nuclear factor erythroid 2-related factor 2 (NRF2), vitiligo, vitiligo index of disease activity (VIDA) score, microRNA-146a (miR-146a), oxidative stress, E3 ubiquitin ligase, keap1, vitexin

## Abstract

Background: Vitiligo, a chronic depigmentation disorder driven by oxidative stress and immune dysregulation, remains poorly understood mechanistically. The Keap1/NRF2/ARE pathway is critical for melanocyte protection against oxidative damage; however, the role of Cullin-3 (CUL3), a scaffold for E3 ubiquitin ligases that regulate NRF2 degradation, and its interplay with inflammatory mediators in vitiligo pathogenesis are underexplored. This study investigates CUL3, NRF2, and the associated regulatory networks in vitiligo, integrating clinical profiling and computational docking to identify therapeutic targets. Methods: A case-control study compared non-segmental vitiligo patients with age-/sex-matched controls. Lesional skin biopsies were analyzed by qRT-PCR for the expression of *CUL3*, *NRF2*, miRNA-146a, *FOXP3*, *NF-κB*, *IL-6*, *TNF-α*, and *P53*. Molecular docking was used to evaluate vitexin’s binding affinity to Keap1, validated by root mean square deviation (RMSD) calculations. Results: Patients with vitiligo exhibited significant downregulation of *CUL3* (0.27 ± 0.03 vs. 1 ± 0.58; *p* = 0.013), *NRF2* (0.37 ± 0.26 vs. 1 ± 0.8; *p* = 0.001), and *FOXP3* (0.09 ± 0.2 vs. 1 ± 0.3; *p* = 0.001), alongside the upregulation of miRNA-146a (4.7 ± 1.9 vs. 1 ± 0.8; *p* = 0.001), *NF-κB* (4.7 ± 1.9 vs. 1 ± 0.5; *p* = 0.001), *IL-6* (2.8 ± 1.5 vs. 1 ± 0.4; *p* = 0.001), and *TNF-α* (2.2 ± 1.1 vs. 1 ± 0.3; *p* = 0.001). *P53* showed no differential expression (*p* > 0.05). Docking revealed a strong binding of vitexin to Keap1 (RMSD: 0.23 Å), mirroring the binding of the control ligand CDDO-Im. Conclusions: Dysregulation of the CUL3/Keap1/NRF2 axis and elevated miRNA-146a levels correlate with vitiligo progression, suggesting a role for oxidative stress and immune imbalance. Vitexin’s high-affinity docking to Keap1 positions it as a potential modulator of the NRF2 pathway, offering novel therapeutic avenues. This study highlights the translational potential of targeting the ubiquitin–proteasome and antioxidant pathways in the management of vitiligo.

## 1. Introduction

Vitiligo is a chronic, acquired skin disorder characterized by the progressive loss of functional melanocytes, resulting in well-demarcated depigmented macules and patches on the skin [1,2]. The global prevalence of vitiligo ranges between 0.5% and 2%, affecting individuals of all ages and ethnic backgrounds [3]. Clinically, vitiligo is classified into two major forms: segmental vitiligo (SV), which presents as localized depigmented areas confined to a specific body segment, and non-segmental vitiligo (NSV), characterized by bilateral and often symmetrical depigmentation distributed across various body sites. Beyond its visible manifestations, vitiligo significantly impairs the patients’ quality of life, self-esteem, and psychosocial well-being and is associated with increased susceptibility to sunburn and potentially skin cancer [4,5].

The etiopathogenesis of vitiligo is complex and multifactorial, involving a convergence of genetic predisposition, autoimmune mechanisms, inflammatory responses, and environmental triggers [6]. Among these, oxidative stress has emerged as a pivotal factor, believed to initiate or amplify melanocyte dysfunction and the subsequent immune-mediated destruction [2]. Understanding the molecular regulatory networks underlying melanogenesis and oxidative stress responses is critical for developing targeted therapies to prevent or reverse melanocyte loss in vitiligo [7].

A central player in the cellular defense against oxidative stress is the nuclear factor erythroid 2-related factor 2 (NRF2) signaling pathway [8]. NRF2 acts as a master regulator of antioxidant gene expression, orchestrating cellular responses to elevated levels of reactive oxygen species (ROS). Under homeostatic conditions, NRF2 is sequestered in the cytoplasm by its repressor, Kelch-like ECH-associated protein 1 (Keap1), which functions as an adaptor for the Cullin-3 (CUL3)-based E3 ubiquitin ligase complex, targeting NRF2 for proteasomal degradation [9]. Upon oxidative challenge, conformational changes in Keap1 lead to the dissociation of the Keap1–NRF2 complex, allowing NRF2 to translocate into the nucleus. There, NRF2 binds to antioxidant response elements (AREs) in the promoters of target genes, inducing the expression of a battery of cytoprotective and detoxifying enzymes [10]. This Keap1/NRF2/ARE axis is thus essential for maintaining cellular redox homeostasis and has been implicated as a promising therapeutic target in vitiligo, where enhanced NRF2 activity may counteract oxidative damage and melanocyte loss [11].

The cullin-RING E3 ubiquitin ligase family, comprising over 200 multi-subunit complexes, is integral to the regulation of protein turnover in mammalian cells [12]. Among its members, CUL3 plays a particularly important role in modulating the stability of proteins involved in oxidative stress and immune responses [13]. Dysregulation of CUL3-mediated ubiquitination may, therefore, contribute to the pathogenesis of vitiligo by altering the degradation dynamics of key regulatory proteins, such as NRF2 [14].

In addition to oxidative stress, inflammatory pathways also contribute to melanocyte destruction in vitiligo [15]. Nuclear factor kappa-light-chain-enhancer of activated B cells (NF-κB) is a redox-sensitive transcription factor that orchestrates inflammatory gene expression and can be activated by ROS such as hydrogen peroxide (H_2_O_2_) [16]. Activation of NF-κB leads to the transcription of proinflammatory cytokines, including interleukin-6 (IL-6) and interleukin-8 (IL-8), which have been implicated in the autoimmune attack on melanocytes. Notably, the NRF2/ARE pathway has been shown to antagonize NF-κB activation, thereby exerting anti-inflammatory effects and further supporting its therapeutic potential in vitiligo [17,18].

The precise mechanisms underlying melanocyte loss in vitiligo remain incompletely understood. Current evidence supports a model in which genetic and epigenetic factors interact with environmental stressors to trigger autoimmune, neural, cytotoxic, and oxidant–antioxidant imbalances, ultimately leading to melanocyte depletion [19]. Melanogenesis is tightly regulated by a network of signaling pathways, microRNAs (miRNAs), and inflammatory mediators [20]. MiRNAs, particularly miRNA-146a, have garnered attention as critical post-transcriptional regulators of gene expression, modulating both innate and adaptive immune responses [21,22]. Regulatory T cells (Tregs), characterized by the expression of the transcription factor Forkhead box protein 3 (Foxp3), are essential for maintaining immune tolerance and preventing autoimmunity. Dysregulation of Tregs and their associated miRNAs, such as miRNA-146a, has been implicated in the pathogenesis of several autoimmune diseases, including vitiligo [23,24]. However, the interplay between miRNA-146a and Foxp3 in vitiligo remains to be fully elucidated.

Emerging evidence suggests that natural compounds with antioxidant and anti-inflammatory properties may offer therapeutic benefits in vitiligo. Vitexin, a flavonoid glycoside found in various medicinal plants, including hawthorn and pearl millet, has demonstrated potent antioxidant and anti-inflammatory activities in preclinical models [25]. Notably, vitexin has been shown to activate the NRF2 signaling pathway, enhance the expression of downstream antioxidant genes such as heme oxygenase-1 (HO-1), and suppress the release of proinflammatory cytokines, including IL-6 and tumor necrosis factor-alpha (TNF-α) [26]. An insufficient activation of NRF2 in the context of oxidative stress has been linked to reduced antioxidant enzyme expression and increased melanocyte vulnerability in vitiligo [27]. Thus, vitexin may hold promise as a modulator of the NRF2 axis and a potential therapeutic agent in vitiligo; however, its precise molecular mechanisms of action in this context remain to be clarified.

In this study, the authors aim to comprehensively profile the expression of CUL3 and key regulatory molecules, including NRF2, NF-κB, miRNA-146a, and Foxp3, in patients with vitiligo, integrating clinical, in silico docking, and bioinformatics analyses. By elucidating the molecular interplay between these pathways and assessing the potential of vitexin as an NRF2 inducer, our findings may provide novel insights into the pathogenesis and therapeutic targeting of vitiligo.

## 2. Materials and Methods

### 2.1. Ethical Statement

This study was conducted in accordance with the ethical standards of the Ethics Review Board at Suez Canal University (approval reference code: 5267#). Written informed consent was obtained from all participants prior to sample and data collection, ensuring the protection of participant rights and confidentiality.

### 2.2. Study Design and Participants

A case-control study design was employed, enrolling 40 eligible patients diagnosed with non-segmental vitiligo and 40 healthy controls between January and August 2024. The inclusion criteria for patients comprised a confirmed clinical diagnosis of non-segmental vitiligo, with no phototherapy or systemic treatment within the preceding two months to minimize the confounding effects of recent therapy on molecular and clinical parameters. Exclusion criteria encompassed any personal, past, or family history of autoimmune disorders, neoplastic diseases, pregnancy, lactation, or other comorbidities potentially affecting immune function.

All participants underwent comprehensive medical evaluations, including detailed history, general and dermatological examinations, and laboratory investigations (complete blood count; erythrocyte sedimentation rate; blood glucose, liver, renal, and thyroid function tests; antithyroid antibodies; antinuclear antibodies; and rheumatoid factor). The dermatological assessment included the classification of vitiligo type, lesion distribution, and extent using the Vitiligo Area Scoring Index (VASI) [28] and the Vitiligo Index of Disease Activity (VIDA) [29]. Wood’s light examination was performed to confirm the diagnosis and exclude subclinical lesions.

### 2.3. Skin Biopsy Collection and Processing

For each patient, a 4 mm punch biopsy was obtained from the lesional skin (adjacent to the inner border of a vitiligo patch). For controls, a 4 mm punch biopsy was collected from the upper back. Specimens were immediately preserved in lysis buffer to stabilize molecular targets and stored at –80 °C until analysis. Non-lesional biopsies were not obtained from vitiligo patients to minimize procedural burden and ethical concerns regarding additional invasive sampling. This approach is consistent with standard practice in molecular studies, where lesional tissue is compared to healthy controls to elucidate disease-specific alterations [30].

### 2.4. Molecular Analyses

#### 2.4.1. RNA Extraction and cDNA Synthesis

Approximately 100 mg of tissue was pulverized under liquid nitrogen. Total RNA was extracted using the RNeasy Mini Kit (Qiagen, Hilden, Germany; cat. no. 74104), following the manufacturer’s protocol [31]. RNA concentration and purity were assessed using a “Nanodrop ND-1000 spectrophotometer (NanoDrop Tech. Inc., Wilmington, DE, USA)” and gel electrophoresis. To preserve RNA integrity, samples were stored in individual aliquots at −80 °C until subsequent analysis.

First-strand cDNA synthesis was performed using the QuantiTect Reverse Transcription Kit (Qiagen, cat. no. 205311) with random hexamer primers, adjusting the final RNA concentration to 0.1 ng/μL. Reverse transcription was performed using the T-Professional Basic PCR system (Biometra, Goettingen, Germany). The reverse transcription protocol consisted of incubation at 16 °C for 30 min, 42 °C for 30 min, and 85 °C for 5 min, followed by a final hold at 4 °C.

#### 2.4.2. Quantitative PCR

The RT products were diluted 1:1 with distilled water before amplification. Quantitative real-time PCR (qRT-PCR) was conducted using the ABI PRISM 7000 Sequence Detection System (Applied Biosystems) and SYBR Green chemistry (Qiagen, Hilden, Germany; cat. no. 204141). The authors followed the “Minimum Information for Publication of Quantitative Real-time PCR Experiments (MIQE)” guidelines for conducting qRT-PCR experiments [32]. Gene-specific primers (Table 1) were used to assess the expression of *CUL3*, *NRF2*, *IL-6*, *P53*, *NF-κB*, *FOXP3*, *TNFα*, and miRNA146a [31]. In each run, appropriate negative controls were applied: “no template (NTC) and no reverse transcriptase (NRT)” controls. PCR cycling conditions included an initial denaturation step at 94 °C for 30 s, followed by annealing at a gene-specific temperature (60–63 °C) and extension at 72 °C for 30 s, repeated for 35 cycles [31]. The housekeeping gene “glyceraldehyde-3-phosphate dehydrogenase (*GAPDH*)” was used as the endogenous control. The calculation of the relative quantity of each quantified transcript to *GAPDH* in patients versus control samples was performed using the following formula: “2^−ΔΔCq^; where ΔΔC_q_  =  (C_q_ *specified gene* −  C_q_ *GAPDH*)_patient_  −  (C_q_ *specified gene* −  C_q_ *GAPDH*)_mean controls_” [33]. The quantification cycle (Cq) is defined as the number of PCR cycles required for the fluorescence signal to exceed a predetermined threshold [32]. Similar calculation steps were performed on miRNA146a normalized to the small nuclear RNU6B expression, after ensuring its consistent and uniform expression across all the samples following MIQE guidelines. Due to sample and resource constraints, additional validation by Northern blotting or small RNA sequencing was not performed; however, qRT-PCR remains the gold standard for miRNA quantification in clinical research and is widely used for miRNA expression analysis in similar studies [34,35].

Inter- and intra-assay coefficients of variation (CV) were calculated to ensure assay precision. Technical triplicates were analyzed for all qRT-PCR assays, and CVs below 10% (intra-assay) and 15% (inter-assay) were maintained as acceptability thresholds, consistent with standardized guidelines for molecular diagnostics [36,37].

#### 2.4.3. Quality Control and Assay Standardization

To minimize batch effects, all molecular assays were performed within a single experimental batch whenever feasible. For assays requiring multiple runs, inter-batch calibration was achieved using standardized reference controls, including identical standard curves and pooled quality control samples to normalize between runs. Samples were collected using standardized protocols, processed within a consistent timeframe, and stored at −80 °C to maintain their integrity. RNA extractions were carried out using the same commercial kits and protocols across all samples by trained personnel to ensure consistency. To reduce operator-dependent variability, molecular assays were conducted either by a single operator or by personnel trained under the same standard operating procedures (SOPs). All measurements were performed in technical triplicate, and data quality was ensured by excluding outliers according to predefined thresholds (e.g., Ct deviation > 0.5 in qPCR replicates).

### 2.5. Bioinformatics and Pathway Enrichment Analyses

#### 2.5.1. Functional Enrichment

Gene ontology (GO) and Kyoto Encyclopedia of Genes and Genomes (KEGG) pathway enrichment analyses were performed using “Database for Annotation, Visualization, and Integrated Discovery” (DAVID) v6.8 (https://davidbioinformatics.nih.gov/) (accessed on 30 August 2024) [38]. GO and KEGG terms with a false discovery rate (FDR) <0.05 were considered significant.

#### 2.5.2. Protein Interaction and Localization

The KEAP1–CUL3 E3 ligase complex and related oxidative stress pathways were analyzed using multiple databases: NCBI Gene (https://www.ncbi.nlm.nih.gov/gene/2735), GeneCards (www.genecards.org), Ensembl (www.ensembl.org), UniProt (https://www.uniprot.org/), and Compartments (https://compartments.jensenlab.org/Search) for subcellular localization. Network Analyst v3.0 (https://www.networkanalyst.ca) was used to identify transcriptional interactors [39], and STRING v11.5 (https://string-db.org) for protein–protein interaction networks [40]. Pathway enrichment was further validated using Reactome. All online resources accessed on 30 August 2024.

### 2.6. Molecular Docking Studies

#### 2.6.1. Protein and Ligand Preparation

The crystal structure of Keap1 (PDB: 4CXT) was retrieved from the RCSB Protein Data Bank. Water molecules were removed, and a single peptide chain was selected using UCSF Chimera v1.16 [41]. Ligand structures, including vitexin, were obtained from NCBI PubChem in SDF format and converted to PDB using PyMOL. DrugBank was referenced for the structural validation of vitexin [31].

#### 2.6.2. Docking Protocol

Molecular docking was performed using AutoDock Tools 4.2. Binding sites were identified with Discovery Studio and Chimera. Ligand energy minimization involved adjusting rotatable bonds and torsion angles, taking into account nonpolar hydrogens. The grid box was set to 40 × 40 × 40 Å, centered at (8.195, –10.01, –15.536), encompassing all relevant binding residues. Docking results were analyzed for binding energies and interaction profiles, with visualization in AutoDock Tools 4.2.

#### 2.6.3. Docking Validation

To ensure methodological rigor, redocking of known ligands was performed to validate the docking protocol, and root mean square deviation (RMSD) values were calculated. RMSD values < 2.0 Å were considered indicative of reliable docking [42]. Binding energies and RMSD values are reported in tabular format.

Docking results showed positive binding energies for CDDO (+192.91 kcal/mol) and vitexin (+13.08 to +14.07 kcal/mol). While such values are atypical, they may reflect limitations of the scoring function or charge distribution rather than true binding inefficacy. Ligand poses exhibited low RMSD values (0.20–0.23 Å), supporting stable docking conformations and suggesting potential binding that warrants further experimental or computational refinement.

#### 2.6.4. In Silico Prediction of Pharmacokinetic and Drug-likeness Properties of Vitexin

It was performed using the SwissADME online tool (www.swissadme.ch) (accessed on 4 July 20, 2025) [43]. Parameters including lipophilicity (logP), solubility, gastrointestinal absorption (GI), cytochrome P450 enzyme inhibition, topological polar surface area (TPSA), drug-likeness filters, and the BOILED-Egg model for passive absorption and BBB penetration were analyzed. Vitexin′s SMILES string was input into the SwissADME platform, and the generated profile was used to evaluate its potential as a drug-like molecule for oral administration.

### 2.7. Power and Statistical Analyses

Power analysis (α = 0.05, two-tailed) indicated that our sample size (n = 40 per group) achieves greater than 80% power to detect moderate effect sizes (Cohen′s d ≥ 0.63) for the primary biomarker endpoint, consistent with published recommendations for molecular biomarker studies [44,45]. Power was calculated only for the prespecified primary endpoint; no multiplicity adjustments were applied to secondary analyses, which are considered exploratory.

The yielded data were tested for normality using the Shapiro–Wilk test (significance threshold: *p* < 0.05). Descriptive statistics are presented as mean ± standard deviation (SD) or median (interquartile range), as appropriate. Categorical variables were compared using the chi-square test. For continuous variables, one-way ANOVA was used for normally distributed data, while the Mann–Whitney U test and the Kruskal–Wallis test were applied for non-normally distributed data. Correlation analyses were performed using Spearman’s rank correlation coefficient. The qRT-PCR results were analyzed using the 2^−[ΔΔCq]^ method, as mentioned in Section 2.4.2., with expression levels interpreted as upregulation (>1) or downregulation (<1) relative to controls. All statistical analyses were conducted using IBM SPSS Statistics version 22.0, with a significance level set at *p* < 0.05.

## 3. Results

### 3.1. Bioinformatics and Pathway Analysis

#### 3.1.1. Keap1–NRF2 Interaction and Oxidative Stress Networks

**Figure 1:**
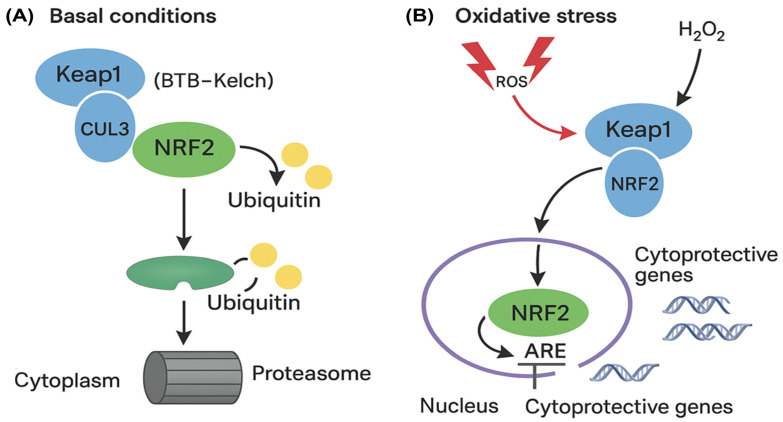
Schematic depiction of the Keap1–NRF2 pathway and its regulation by oxidative stress. (**A**) In baseline settings (left), Keap1 associates with CUL3 to constitute an E3 ubiquitin ligase, facilitating the ubiquitination and subsequent proteasomal degradation of NRF2, thereby sustaining diminished NRF2 levels in the cytoplasm. (**B**) Exposure to oxidative stress results in the modification of cysteine residues on Keap1 by reactive oxygen species (ROS), hence affecting its interaction with NRF2. This facilitates the accumulation of NRF2, its translocation to the nucleus, and its binding to antioxidant response elements (AREs), resulting in the transcriptional activation of cytoprotective and antioxidant genes. Ub: Ubiquitin; ARE: Antioxidant Response Element.

Functional annotation revealed that Keap1 (a redox-sensitive BTB-Kelch protein) forms a CUL3-dependent E3 ligase complex that degrades NRF2 under basal conditions. Oxidative stress disrupts this interaction, allowing NRF2 to accumulate and activate cytoprotective genes (Figure 1A,B).

**Figure 2:**
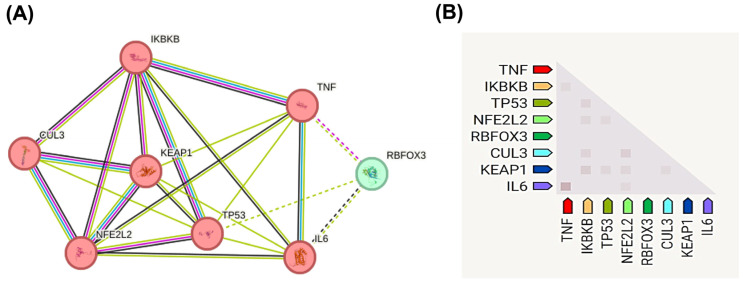
Protein–protein interaction (PPI) network and co-expression analysis of oxidative stress-related genes in vitiligo. (**A**) PPI network shows functional associations among key oxidative stress and inflammatory regulators. Node colors represent different functional clusters: proinflammatory mediators (pink/red nodes: TNF, IKBKB, TP53, NFE2L2, IL6, CUL3, KEAP1) and regulatory factors (green node: RBFOX3). Edge thickness indicates interaction confidence scores, with thicker lines representing stronger evidence-based associations. The network demonstrates two distinct clusters with an average k-means clustering coefficient of 0.838 and statistically significant interactions (*p* = 0.029). Line colors in the network represent different sources of evidence for the associations depicted: purple: experimentally determined interactions; green: associations derived from curated databases; blue: associations identified through text mining; and black: co-expression relationships. The solid and dashed lines indicate direct and indirect evidences, respectively. (**B**) Co-expression heatmap displaying the relative expression levels of the same genes, with color intensity indicating the magnitude of co-expression. Data were generated using the STRING database v11.5 (https://string-db.org) (accessed 30 August 2024), with a minimum required interaction score of 0.4 (medium confidence). Clustering analysis reveals the central roles of CUL3, KEAP1, and NFE2L2 (NRF2) in coordinating cellular responses to oxidative stress, supporting their dysregulation in vitiligo pathogenesis.

Protein–protein interaction (PPI) analysis identified two clusters (k-means coefficient: 0.838, *p* = 0.029), with CUL3 and NRF2 co-expressed in stress-response networks (Figure 2A,B).

#### 3.1.2. CUL3 Structural and Regulatory Networks

**Figure 3:**
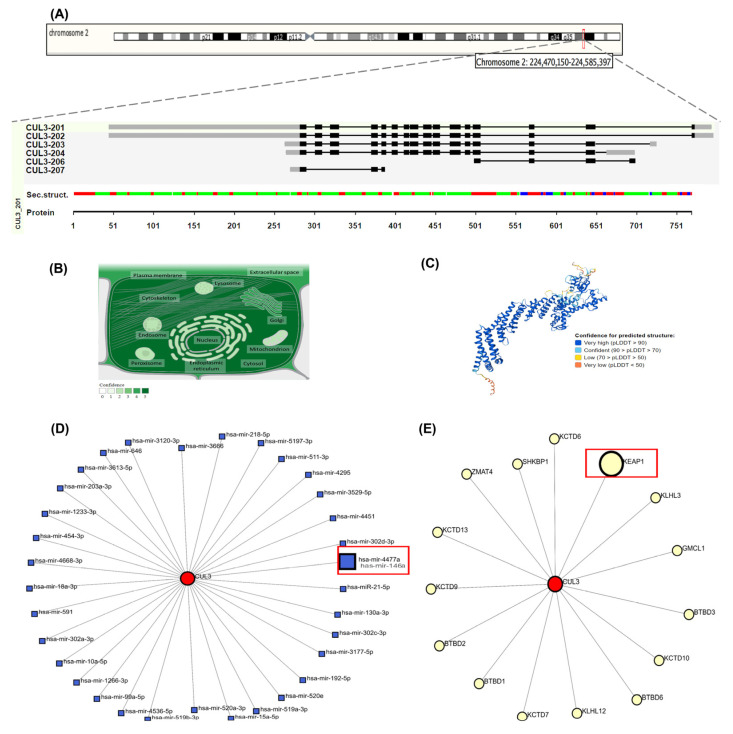
Comprehensive structural and functional characterization of CUL3 (Cullin-3) gene and protein. (**A**) Genomic organization and chromosomal localization of CUL3 on chromosome 2q36.2 (coordinates: 224,470,150–224,585,397, GRCh38/hg38 assembly), spanning 115,248 base pairs on the minus strand. The gene contains six alternative transcripts (CUL3-201 through CUL3-207), with an exon–intron structure depicted as black boxes (exons) and connecting lines (introns). Secondary structure prediction shows α-helices (green), β-strands (red), and β-sheets (blue) along the protein sequence (amino acids 1–751). (**B**) Subcellular localization of CUL3 protein across cellular compartments, with color intensity reflecting confidence levels of experimental evidence. CUL3 shows the highest expression in the nucleus, cytoskeleton, plasma membrane, and cytoplasm, consistent with its role as a scaffold protein in E3 ubiquitin ligase complexes. (**C**) Three-dimensional protein structure prediction of CUL3-201 isoform generated using AlphaFold v2.3.2, with confidence scores indicated by color coding. (**D**) MicroRNA regulatory network showing 29 miRNAs predicted to target CUL3 mRNA (blue squares), including miR-146a and miR-3024-3p highlighted in red boxes, which are implicated in oxidative stress responses. (**E**) The protein–protein interaction network displays 29 experimentally validated CUL3-interacting proteins (yellow circles), with KEAP1 highlighted in a red box due to its central role in NRF2 regulation. Network data were obtained from NetworkAnalyst v3.0 and STRING databases (accessed on 30 August 2024), representing functional associations in cellular processes, such as protein ubiquitination, oxidative stress response, and cell cycle regulation.

CUL3 (ENSG00000036257, Chr2q36.2) (Figure 3A) encodes a protein that is localized primarily to the nucleus, cytoskeleton, and plasma membrane (Figure 3B), with a high-confidence predicted structure illustrated in Figure 3C. NetworkAnalyst identified 29 interacting proteins (e.g., ubiquitination regulators) (Figure 3D) and six miRNAs linked to oxidative stress (Figure 3E).

### 3.2. Molecular Docking and Ligand Interactions

Proteins and ligands were retrieved from PubChem in structure data file (SDF) format, then processed using SwissADME (http://www.swissadme.ch/) (accessed 5 July 2025) to evaluate its pharmacokinetic properties. Table 2 presents the compounds that were evaluated following the adjustment of bond order, the addition of hydrogens (with nonpolar hydrogens concealed), and energy minimization. The first compound listed is the well-known CDDO-Im, while the second one is vitexin, presented in various conformers.

#### 3.2.1. Visualization of Receptor Binding Site and Analysis of Ligand–Receptor Interactions

Various preceding virtual screening and molecular docking research showed the activity of some drug products against the clinical targets of Keap1 protein as anti-inflammatory and antidrug, like CDDO-Im. Among those, one suggested that vitexin can modulate the inflammatory response by stimulating the NRF2/ARE signaling pathway, indicating its potential role as an NRF2 activator in vitiligo. Nevertheless, the precise mechanism through which vitexin regulates the NRF2/ARE pathway in vitiligo remains under investigation [26].

- CDDO

**Figure 4:**
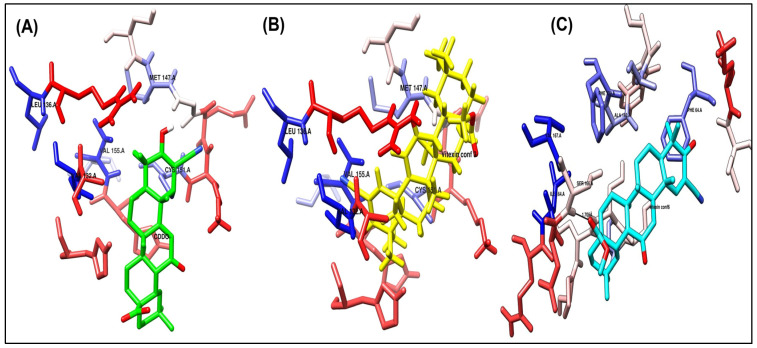
Binding disposition of docked compounds CDDO, Vitexin conformer 3, and Vitexin conformer 6 (**A**–**C**), respectively, inside the 4CXT binding site.

**Figure 5:**
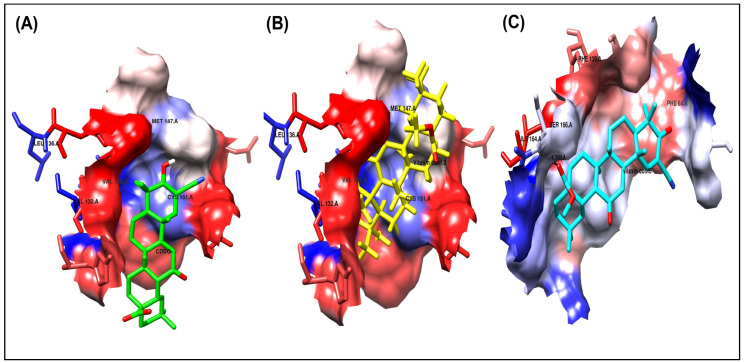
Ligand–receptor interactions of docked compounds CDDO, Vitexin conformer 3, and Vitexin conformer 6 (**A**–**C**), respectively, inside the 4CXT binding site. The interaction of docked compounds with lipophilic characteristics, in which the amino acids highlighted in red represent the hydrophilic amino acids.

As shown in Figure 4A and Figure 5A, the co-crystallized ligand CDDO interacts within the binding site pocket of 4CXT protein with lipophilic interactions (dipole–dipole interactions) with Cystine 151, Methionine 147, Valine 123, Valine 155, and Leucine 136.

- Vitexin conformer 3

It has the same better binding points as CDDO, as it made the same interactions within the binding site pocket of 4CXT protein with lipophilic interactions (dipole–dipole interactions) with Cystine 151, Methionine 147, Valine 123, Valine 155, and Leucine 136. (Figure 4B and Figure 5B).

- Vitexin conformer 6

It also interacts with Keap1, but through different sites, via one hydrogen bond with Serine 190, at a distance of 1.7 Å. Figure 4C and Figure 5C also show dipole–dipole interactions with Phenylalanine 64, Alanine 140, Phenylalanine 139, Isoleucine 164, and Valine 167 residues.

A summary of the interactions between the ligands and the receptors for the docked compounds, as determined by AutoDock, focusing solely on the receptor binding site and the key amino acid residues involved, is presented in Table 3.

#### 3.2.2. Self-Docking Results

The outcomes were assessed by determining the root-mean-square deviation (RMSD) for each ligand and target, comparing the reference position of the known CDDO ligand with the docked position predicted by various docking calculations [46]. The RMSD analysis was conducted utilizing the RMSD analysis software included in Autodock Tools version 1.5.7. Self-docking serves as a method to evaluate the reliability of the software results, as illustrated in Table 4. Docking software that yields RMSD values below two is considered optimal for docking compounds within the receptor binding site.

**Figure 6:**
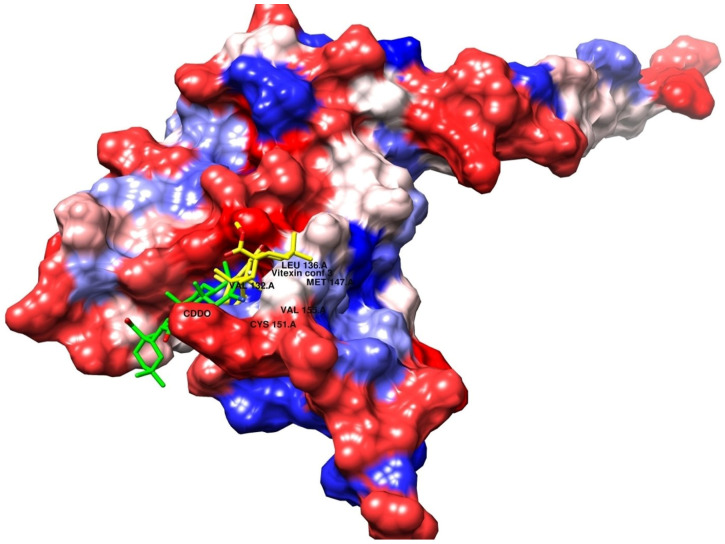
Superimposed poses of the output docked tested compound (vitexin, yellow) and the co-crystallized ligand (CDDO, green) inside the binding sites of the 4CXT receptor.

Upon overlaying the tested compounds, it was observed that they were positioned within the same pocket as the co-crystallized ligand, as illustrated in Figure 6, and that they bind relatively to the same site in the receptor pocket.

#### 3.2.3. In Silico ADME Profiling of Vitexin

**Figure 7:**
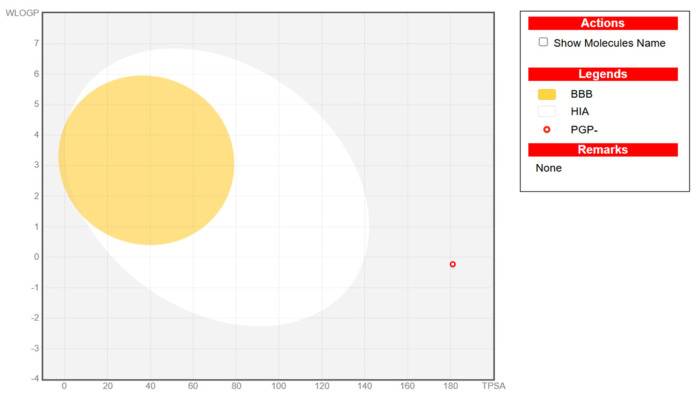
Representative BOILED-Egg plot for vitexin. It illustrates the predicted gastrointestinal absorption (HIA), blood–brain barrier (BBB) penetration, and P-glycoprotein (PGP) substrate status of the studied compound based on its physicochemical properties. The x-axis denotes topological polar surface area (TPSA), and the y-axis represents WLOGP (lipophilicity). The yellow region indicates the physicochemical space associated with high BBB permeability, while the white region represents high probability of passive human intestinal absorption (HIA). The open blue circle denotes compounds predicted as PGP substrates (PGP+), and the open red circle indicates non-substrates (PGP–). The plotted compound (red circle) falls outside both the BBB and HIA regions, suggesting a low probability of central nervous system penetration and limited gastrointestinal absorption.

To assess the drug-likeness and oral availability of vitexin, SwissADME analysis was performed (Supplementary Table S1). Although the compound exhibited low predicted gastrointestinal absorption and limited BBB permeability (as confirmed by the BOILED-Egg model; Figure 7), it showed excellent solubility and a clean metabolic profile, with no predicted inhibition of major cytochrome P450 enzymes. Importantly, vitexin did not trigger Pan-assay Interference Compound (PAINS) or Brenk alerts, suggesting a low risk of promiscuous or toxic behavior.

While vitexin violated certain drug-likeness rules, such as Lipinski (due to high polarity and multiple hydrogen bond donors), such characteristics are common among polyphenolic natural compounds and do not preclude biological activity. Moreover, these limitations can be mitigated through modern formulation strategies.

### 3.3. Cohort Characteristics

The study included 40 patients with vitiligo and 40 controls (mean age: 30 ± 3 years vs. 30 ± 2 years). No significant differences were observed regarding age (*p* = 0.919) or sex (52.5% male vs. 55%, *p* = 0.5).

The clinical data of the affected group is shown in Table 5, revealing that the mean duration of the appearance of affected areas with vitiligo was 3 ± 1.5 months, and the mean number of warts was 8 ± 6 warts. The order of affected regions in the genital tract by warts was penis only, penis and scrotum, groin and penis, groin only, and groin, scrotum, and penis regions, respectively. The onset of warts was gradual in 66.6% of patients, with a stationary course in 17%, and the majority exhibited a morphological feature of papules. Wart recurrence occurred in 6.6% of patients after a complete cure.

### 3.4. Molecular Profiling

**Figure 8:**
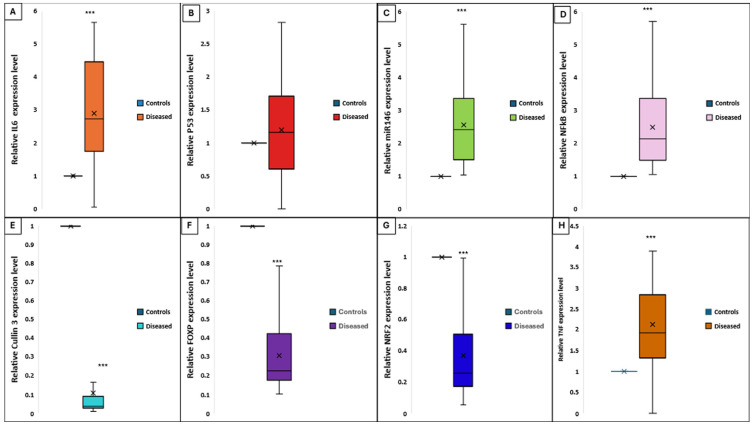
Differential expression levels of key genes and cytokines in lesional skin of vitiligo patients versus healthy controls. Each box represents the interquartile range (IQR), the horizontal line within the box indicates the median, and the “×” denotes the mean. Whiskers indicate the range. (**A**) IL-6, (**B**) P53, (**C**) miR-146a, (**D**) NF-κB, (**E**) CUL3, (**F**) FOXP3, (**G**) NRF2, and (**H**) TNF-α in diseased (vitiligo) and control groups. Expression was quantified by qRT-PCR and normalized to endogenous controls. Significant upregulation of IL-6, miR-146a, NF-κB, and TNF-α and significant downregulation of CUL3, NRF2, and FOXP3 were observed in patients with vitiligo compared to controls (*** *p* < 0.001). No significant difference was detected for P53 expression.

The relative expression levels of *CUL3* and *NRF2* were significantly downregulated in vitiligo patients compared to healthy controls, with mean ± SD values of 0.27 ± 0.03 and 0.37 ± 0.26, respectively, versus 1 ± 0.58 (*p* = 0.013). In contrast, miR-146a exhibited marked upregulation in vitiligo tissues (4.8 ± 1.9 vs. 1 ± 0.8; *p* = 0.001). Concurrently, *FOXP3* expression was significantly reduced in patients (0.089 ± 0.2 vs. 1 ± 0.3; *p* = 0.001). Pro-inflammatory mediators were also elevated: *NF-κB* (4.7 ± 1.9 vs. 1 ± 0.5; *p* = 0.001), *IL-6* (2.8 ± 1.5 vs. 1 ± 0.4; *p* = 0.001), and *TNF-α* (2.2 ± 1.1 vs. 1 ± 0.3; *p* = 0.001). No significant difference was observed in P53 expression between groups (*p* > 0.05) (Figure 8).

### 3.5. Correlation of CUL3 and miRNA-146a Expression with Vitiligo Disease Activity

**Figure 9:**
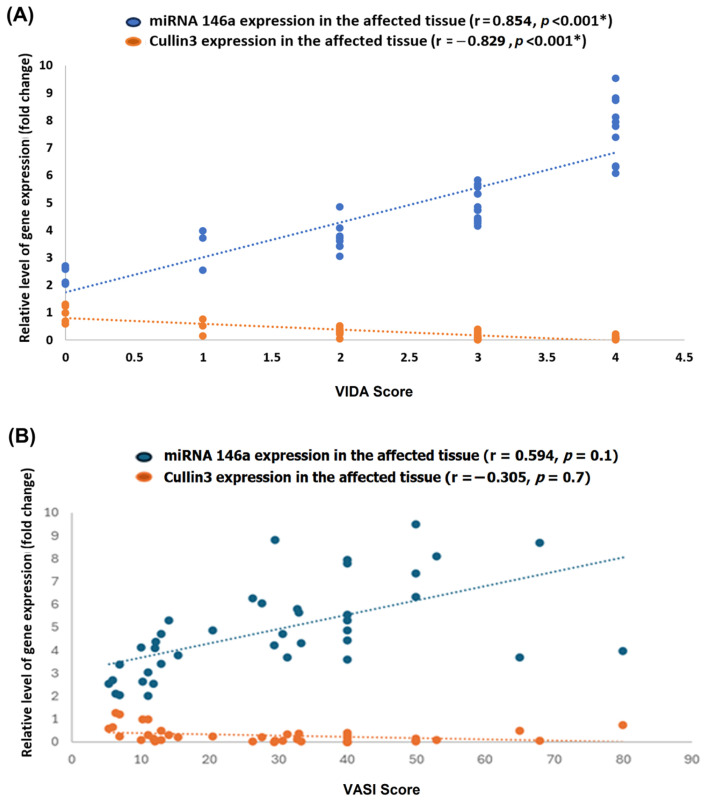
Correlation of miRNA-146a and CUL3 expression with Vitiligo Disease Activity (VIDA) and Vitiligo Area Scoring Index (VASI). Scatter plots illustrate the relationship between the relative expression levels (fold change) of miRNA-146a (blue) and CUL3 (orange) in lesional skin and the VIDA score (**A**) or the VASI score (**B**) among patients with vitiligo. The dotted lines represent linear regression fits for each gene. Each point represents an individual patient sample. * Statistical significance was set at a *p*-value of less than 0.05. r: correlation coefficient.

There was a notable positive correlation between miRNA-146a expression levels and the VIDA score (r = 0.854, *p* < 0.001), indicating that higher miRNA-146a expression is associated with increased disease activity. In contrast, CUL3 expression demonstrated a strong negative correlation with the VIDA score (r = −0.829, *p* < 0.001), suggesting that lower CUL3 expression is associated with more active disease (Figure 9A). Otherwise, there were no significant correlations between the above biomarkers and the VASI score (Figure 9B).

### 3.6. Correlation Matrix

**Figure 10:**
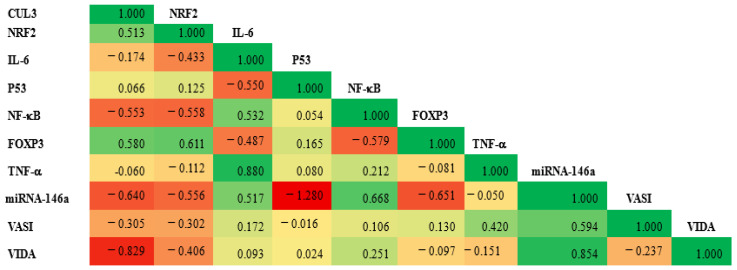
The correlation matrix illustrates the relationship between the expression level of CUL3 and various study parameters. The green color represents a positive correlation, while the red denotes a negative one. The value of r reflects the strength of the correlation. A significance level of *p* < 0.05 was established.

Notably, the CUL3 expression level was moderately positively correlated with the NRF2 and FOXP3 expression (r = 0.513 and 0.580, respectively) and moderately negatively correlated with NF-KB and miRNA146a (r = −0.553 and −0.640, respectively). Additionally, a marked negative correlation was observed between the VIDA score and CUL3 (r = −0.829). At the same time, a strong positive correlation was observed between the VIDA score and miRNA (r = 0.854), as shown in Figure 10. Additionally, a moderate positive correlation was observed between CUL3 and the VASI score.

### 3.7. Mean of Relative Cytokine Expression Levels in Study Groups

**Figure 11:**
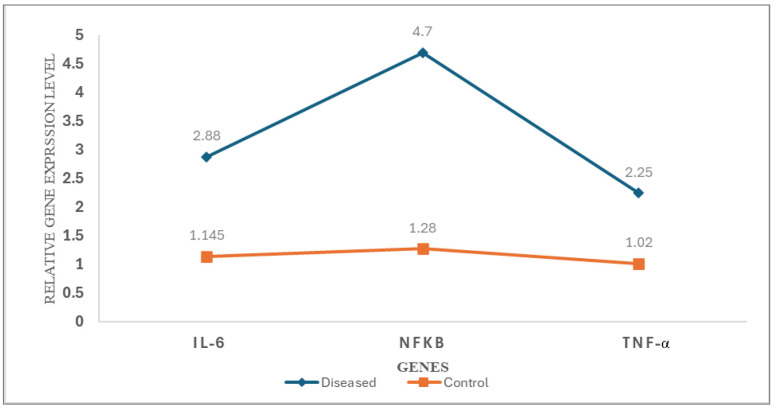
Differential expression profiles of proinflammatory cytokines and transcription factors in patients with vitiligo versus healthy controls. Line graph illustrating the relative expression levels of IL-6, NF-κB, and TNF-α in lesional skin biopsies from vitiligo patients (blue line, diamond markers) compared to healthy controls (orange line, square markers). Gene expression was quantified by qRT-PCR using the 2^(−ΔΔCq)^ method and normalized to *GAPDH* as an endogenous control. Each data point represents the mean relative expression level (n = 40 per group).

The average expression levels of *IL-6*, *NF-κB*, and *TNFα* in the patient group were markedly elevated compared to the control group: IL-6 (2.88-fold vs. 1.145-fold), NF-κB (4.7-fold vs. 1.28-fold), and TNF-α (2.25-fold vs. 1.02-fold) (all *p* < 0.001), as illustrated in Figure 11.

## 4. Discussion

This study provides a comprehensive molecular and bioinformatics analysis of the CUL3 E3 ubiquitin ligase complex and its regulatory partners in vitiligo, integrating clinical, gene expression, and in silico docking data to elucidate the mechanisms underlying melanocyte loss and immune dysregulation. Our principal findings are as follows: (1) significant downregulation of CUL3 and NRF2 in vitiligo tissues compared to controls; (2) upregulation of miRNA-146a, NF-κB, IL-6, and TNF-α, with concurrent downregulation of FOXP3; (3) strong correlations between CUL3, NRF2, FOXP3, and disease activity; and (4) molecular docking supporting the potential of vitexin as an NRF2 pathway modulator. These results collectively support the hypothesis that impaired oxidative stress response and immune imbalance are central to vitiligo pathogenesis.

### 4.1. Oxidative Stress and the Keap1/CUL3/NRF2 Axis in Vitiligo

The pivotal role of oxidative stress in vitiligo is well established, with both exogenous (UV radiation, chemicals) and endogenous (mitochondrial metabolism, immune activation) factors contributing to excessive reactive oxygen species (ROS) production and melanocyte vulnerability [2,47]. The study’s findings of reduced CUL3 and NRF2 expression in vitiligo tissues align with the concept that the Keap1/CUL3/NRF2 axis is disrupted, impairing the cellular antioxidant response and predisposing melanocytes to oxidative damage.

The literature, however, presents some conflicting reports regarding NRF2 function in vitiligo. Most studies demonstrate that NRF2 activation is impaired in vitiligo melanocytes, as evidenced by reduced nuclear translocation and decreased transcriptional activity in response to oxidative stress. This leads to diminished expression of downstream antioxidant enzymes, such as HO-1, and contributes to increased melanocyte susceptibility to oxidative injury and cell death [47,48,49]. In addition, abnormalities in NRF2 localization and function, as well as genetic polymorphisms, have been reported in patients with vitiligo, further supporting a model of impaired NRF2–ARE signaling in disease pathogenesis [49,50].

Conversely, some reports have described the upregulation of NRF2 and its downstream targets in vitiligo skin, which may reflect a compensatory response to chronic oxidative stress or methodological differences in sample type, disease stage, or detection methods [2,51]. Pharmacological studies also show that NRF2 can be therapeutically activated (e.g., by dimethyl fumarate), and such activation confers protection to both normal and vitiligo melanocytes, suggesting that the pathway remains at least partially responsive and may be modulated in a context-dependent manner [48]. However, the downregulation of both CUL3 and NRF2 observed in our cohort suggests a failure of this compensatory mechanism, which may be particularly relevant in active disease.

CUL3, as a scaffold protein for the E3 ubiquitin ligase complex, orchestrates the proteasomal degradation of NRF2 under basal conditions. Upon oxidative stress, Keap1 is inactivated, allowing NRF2 to accumulate and translocate to the nucleus, where it induces the transcription of cytoprotective genes. Our co-expression and pathway analyses reinforce the centrality of this axis in maintaining melanocyte homeostasis and highlight its disruption as a driver of vitiligo pathogenesis.

In summary, while the predominant body of evidence supports impaired NRF2 activation as a key pathogenic mechanism in vitiligo, compensatory or context-dependent NRF2 responses have also been described. These may represent attempts by melanocytes to counteract ongoing oxidative stress but appear insufficient to restore redox balance and prevent cell loss in most patients [27,47,48,49]. Further research is needed to clarify the precise regulatory mechanisms and therapeutic implications of NRF2 modulation in vitiligo.

### 4.2. Immune Dysregulation: miRNA-146a, FOXP3, and Cytokine Networks

This study further demonstrates the significant upregulation of miRNA-146a and proinflammatory mediators (NF-κB, IL-6, and TNF-α), accompanied by the downregulation of Foxp3 in vitiligo tissues. These findings are in line with the growing body of evidence implicating immune dysregulation and chronic inflammation in vitiligo [52,53]. miRNA-146a is recognized as a key regulator of immune responses, modulating NF-κB signaling and cytokine production [54]. The observed upregulation of miRNA-146a in the present cohort and its positive correlation with disease activity (VIDA score) and NF-κB support its role in promoting inflammation and disease progression. This is consistent with previous studies reporting elevated miRNA-146a in vitiligo patients [55,56], though some discrepancies exist, potentially due to differences in sample type (tissue vs. blood) or patient characteristics [52,57].

Foxp3, the master transcription factor for regulatory T cells (Tregs), is essential for maintaining immune tolerance and preventing autoimmunity [58]. The marked downregulation of Foxp3 observed in our study is in agreement with prior reports of reduced Treg function in vitiligo [59,60]. This reduction likely contributes to the breakdown of immune tolerance and the selective targeting of melanocytes. Interestingly, some studies have reported an increased Foxp3+ Treg infiltration in lesional skin, suggesting compensatory recruitment to the sites of inflammation [61]. Our findings of a negative correlation between Foxp3 and both disease activity and miRNA-146a further underscore the interplay between immune regulation and oxidative stress in vitiligo.

In addition to VIDA, we explored the relationship between molecular markers and disease severity using VASI. A moderate but non-significant positive correlation was observed between miRNA-146a expression and VASI (r = 0.594, *p* = 0.1), suggesting a possible association between higher miRNA-146a levels and more extensive depigmentation. Conversely, CUL3 expression showed a weak negative correlation with VASI (r = –0.305, *p* = 0.7), which may reflect the proposed oxidative stress-related impairment of the Keap1–NRF2–CUL3 pathway in vitiligo. Although these findings did not reach statistical significance, they might be biologically relevant and consistent with the proposed roles of these biomolecules in inflammation and redox imbalance. Further studies with larger cohorts are warranted to confirm these associations.

The elevated levels of proinflammatory cytokines, particularly TNF-α and IL-6, in the current study patients support previous reports linking these mediators to vitiligo pathogenesis and activity [62,63,64,65,66]. These cytokines exacerbate melanocyte damage, perpetuate local inflammation, and may serve as biomarkers of disease activity.

### 4.3. Integrative Pathogenesis Model and Therapeutic Implications

The observed correlations between CUL3, NRF2, FOXP3, miRNA-146a, and clinical parameters suggest a tightly interconnected network linking oxidative stress, immune dysregulation, and disease progression. Specifically, the negative correlation between CUL3 and VIDA score, as well as the positive correlation between miRNA-146a and VIDA score, highlights their potential as biomarkers of disease activity and therapeutic response. These findings support a model in which impaired CUL3/NRF2 signaling diminishes antioxidant defenses while upregulated miRNA-146a and reduced Foxp3 promote a proinflammatory milieu, collectively driving melanocyte destruction.

Our in silico docking studies suggest that vitexin, a naturally occurring flavonoid, may modulate the NRF2 pathway by directly interacting with the Keap1 receptor, potentially disrupting the Keap1–CUL3–NRF2 complex and thereby enhancing NRF2 stabilization and nuclear translocation. Although the binding affinity of vitexin (+13.08 to +14.07 kcal/mol) is lower than that of the synthetic activator CDDO-Im (+192.91 kcal/mol), vitexin exhibits stable binding conformations (RMSD < 0.23 Å) and forms key hydrogen bonds and lipophilic interactions with residues such as Ser166 and Leu136. These contacts suggest a plausible mechanism of partial Keap1 inhibition.

This computational prediction is supported by experimental evidence showing that vitexin activates the MAPK–Nrf2/ARE pathway in human melanocytes exposed to oxidative stress, leading to reduced apoptosis and reactive oxygen species (ROS) levels and increased expression of antioxidant genes, such as HO-1 and SOD [26]. Notably, these effects are reversed upon Nrf2 knockdown, underscoring the centrality of this pathway in mediating vitexin’s cytoprotective effects [26]. Additionally, in vivo studies in other oxidative stress-related disease models (e.g., pulmonary and renal injury) have confirmed the biological relevance of NRF2 activation by vitexin [67,68].

Taken together, our docking data provide mechanistic support for these biological observations and highlight vitexin as a promising natural NRF2 modulator. While further functional validation is required, particularly in animal models of vitiligo, these findings support the potential of vitexin as an adjunctive therapeutic agent in conditions characterized by oxidative stress and immune dysregulation.

However, the clinical translation of vitexin requires careful consideration of its bioavailability and safety. Vitexin is known to have poor oral bioavailability, primarily due to its low water solubility, limited gastrointestinal absorption, rapid metabolism, and fast systemic clearance. Pharmacokinetic studies in animal models have demonstrated that, after oral administration, vitexin exhibits low absolute bioavailability (approximately 5%) and is rapidly eliminated from plasma. These pharmacokinetic limitations represent a significant barrier to the therapeutic development of this compound. Also, its pharmacokinetic profile presents challenges for systemic delivery. Studies have demonstrated that vitexin-loaded zein/pectin nanoparticles significantly improved bioavailability and stability, with enhanced intestinal uptake in animal models [69]. Similarly, a formulation using nanosuspensions prepared via antisolvent precipitation and high-pressure homogenization notably increased the vitexin dissolution rate compared to the raw compound [70]. These findings suggest that although native vitexin may have low gastrointestinal absorption and poor blood–brain barrier permeability due to its high polarity and extensive hydrogen bonding (as predicted via SwissADME), its bioavailability can be greatly enhanced through advanced drug delivery systems, such as liposomes, nanoparticles, micelles, and phytosomes, which have been shown to enhance the solubility, stability, and bioavailability of vitexin in preclinical models. Such strategies hold promise for improving the systemic exposure and therapeutic efficacy of vitexin in future clinical trials.

Regarding safety, no PAINS or Brenk alerts were triggered in computational screening, indicating a low risk of off-target interference or structural toxicity. Well-designed phase I/II clinical trials are needed to rigorously assess the safety, pharmacokinetics, and efficacy of vitexin in humans before it can be recommended for therapeutic use.

### 4.4. Study Limitations and Future Directions

This study has some limitations. The modest sample size and recruitment from a single ethnic background may limit the generalizability of our findings. Additionally, although we applied strict inclusion and exclusion criteria and recruited participants from the same geographic region and time frame to minimize confounding, we did not systematically quantify factors such as stress, diet, sun exposure, skin site differences, or subclinical inflammation in controls. These factors may contribute to variability in oxidative and immune markers, representing potential sources of bias. As a result, residual confounding from these variables cannot be completely ruled out. Future studies should incorporate standardized assessments, including detailed recording of skin biopsy sites, objective measures of sun exposure, and screening for subclinical inflammation in controls, along with multivariate analyses to further control for their effects. The cross-sectional design precludes the assessment of causality or temporal changes in gene expression associated with disease progression or treatment response. Consequently, we were unable to evaluate whether the changes in CUL3 or NRF2 expression are dynamically linked to therapeutic outcomes in vitiligo patients over time. Longitudinal studies that incorporate serial sampling before, during, and after treatment would be essential to determine whether modulation of these molecular pathways correlates with clinical improvement, as recommended in recent translational and clinical research frameworks for vitiligo [71,72]. Such data would provide stronger evidence for the utility of CUL3 and NRF2 as predictive or pharmacodynamic biomarkers in therapeutic trials.

Furthermore, our study relied on qRT-PCR for gene expression profiling and did not include protein-level validation of vitexin’s effects on the Keap1/NRF2 pathway. Future studies should incorporate independent methods, such as Western blotting or immunohistochemistry, to confirm these findings and address potential post-transcriptional regulation. Additionally, although miRNA-146a expression was measured using qRT-PCR in accordance with the MIQE guidelines, we did not perform orthogonal validation using Northern blotting or small RNA sequencing. Although qRT-PCR is widely accepted for miRNA quantification, future studies should consider incorporating additional validation methods to substantiate these findings further.

Regarding the molecular docking studies, while they provide valuable mechanistic insights, functional validation in primary melanocyte cultures and animal models is warranted to confirm the therapeutic potential of vitexin and other NRF2 activators. We also acknowledge that our study did not include direct or indirect biochemical assessment of oxidative stress, such as measurement of ROS levels or oxidative damage biomarkers (e.g., malondialdehyde, 8-OHdG, protein carbonyls) in clinical samples. This was due to both methodological and resource constraints, as direct ROS measurement in human tissues is technically challenging and often unreliable.

Future research should focus on

- Larger, multi-center cohorts and independent validation to confirm and extend the current findings.
- Longitudinal studies tracking dynamic changes in CUL3/NRF2/miRNA-146a/FOXP3 expression in relation to disease activity and therapeutic interventions.
- Clinical trials evaluating the efficacy of antioxidant and immunomodulatory therapies targeting the studied pathway.
- Incorporate comprehensive oxidative stress profiling, using both direct and validated surrogate biomarkers, to strengthen the mechanistic links between gene expression changes and redox imbalance in vitiligo.
- Multi-omics approach integrating transcriptomic, proteomic, and metabolomic data to unravel the complex interplay of genetic, epigenetic, and environmental factors in vitiligo pathogenesis.

Finally, although vitexin was selected for docking based on prior evidence of NRF2 activation and cytoprotective effects in melanocyte models, the current study did not include comparative benchmarking against other established NRF2 activators, such as sulforaphane, bardoxolone, or curcumin, as well as unrelated flavonoids serving as negative controls. Such analyses would provide valuable context regarding relative binding affinity, docking pose stability, and pharmacophoric characteristics. Additionally, ex vivo functional assays with vitexin on patient-derived samples, directly comparing the molecular responses to clinical severity and activity scores, are recommended. Additionally, functional experiments involving NRF2 knockdown or inhibition (e.g., using siRNA or pharmacological inhibitors) in melanocyte cultures are warranted to test whether the protective effects of vitexin are directly NRF2-dependent. These approaches would provide stronger translational evidence for the therapeutic potential and mechanism of vitexin in vitiligo.

## 5. Conclusions

In summary, the present integrative analysis highlights the central roles of CUL3, NRF2, miRNA-146a, and Foxp3 in the pathogenesis of vitiligo, linking oxidative stress and immune dysregulation to melanocyte loss. The identification of vitexin as a potential modulator of the NRF2 pathway offers new avenues for targeted therapy. These findings advance our understanding of vitiligo and underscore the need for further translational research to develop effective, mechanism-based treatments.

## Figures and Tables

**Table 1:** Primer sequences for qPCR analysis.

Gene	Primers Sequences	Gene ID	Annealing Temperature
*Cullin-3*	Upper: 5′-TCGACAGCTCACACTCCAGCAT-3′ Lower: 5′-GTGCTTCCGTGTATTAGAGCCAG-3′	8452	60 °C
*NRF 2*	Upper: 5′-CACATCCAGTCAGAAACCAGTGG-3′ Lower: 5′-GGAATGTCTGCGCCAAAAGCTG-3′	4780	60 °C
*miRNA 146*	Upper: 5′-GAGAACTGAATTCCATGG-3′ Lower: 5′-GAACATGTCTGCGTATCTC-3′	406938	60 °C
*NF-κB*	Upper: 5′-TGAACCGAAACTCTGGCAGCTG-3′ Lower: 5′-CATCAGCTTGCGAAAAGGAGCC-3′	5970	60 °C
*GAPDH*	Upper: 5′- AGGGCCCTGACAACTCTTTT-3′ Lower: 5′- GATTCAGTGTGGTGGGGGAC-3′	2597	60 °C
*IL-6*	Upper: 5′-AGACAGCCACTCACCTCTTCAG-3′ Lower: 5′-TTCTGCCAGTGCCTCTTTGCTG-3′	3569	60 °C
*FOXP3*	Upper: 5′-GGCACAATGTCTCCTCCAGAGA-3′ Lower: 5′-CAGATGAAGCCTTGGTCAGTGC-3′	50943	61 °C
*TNFα*	Upper: 5′-CTCTTCTGCCTGCTGCACTTTG-3′ Lower: 5′-ATGGGCTACAGGCTTGTCACTC-3′	7124	62 °C
*P53*	Upper: 5′-CCTCAGCATCTTATCCGAGTGG-3′ Lower: 5′-TGGATGGTGGTACAGTCAGAGC-3′	7157	63 °C
*RNU6B*	Upper: 5′-CTCGCTTCGGCAGCACAT-3′ Lower: 5′-TTTGCGTGTCATCCTTGCG-3′	26827	60 °C

**Table 2:** Compound evaluation.

Compound Name	Two-Dimensional Depiction	Three-Dimensional Depiction
CDDO-Im	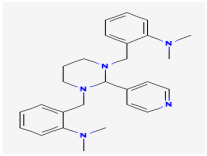	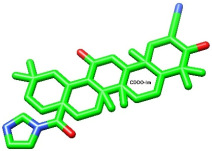
Vitexin	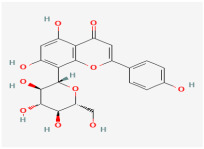	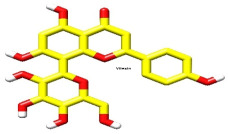

The optimized, energy-minimized structures of potential compounds are presented in both two-dimensional and three-dimensional formats (generated using ChemDraw Pro 8.0 and Discovery Studio software 4.5).

**Table 3:** A summary of the interactions between ligands and receptors for the docked compounds.

Molecular Target and PDB Code	Compound	Hydrogen Bond Analysis			Amino Acids Involved in the Lipophilic Analysis
		No	Hydrogen Bond Ligand/Receptor	Distance (Å)	
4CXT	CDDO				Cyst 151, Meth 147, Val 123, Val 155, and Leu 136.
	Vitexin Conformer 3				Cyst 151, Meth 147, Val 123, Val 155, and Leu 136.
	Vitexin Conformer 6		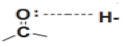	-Ser 1661.7 Å	Phen 64, Ala 140, Phen 139, Ileu 164, and Val 167.

**Table 4:** RMSD values of the best docked ligand conformers.

Result Analysis Software Auto Dock	CDDO Conformer 1	Vitexin	
		Conformer 3	Conformer 6
RMSD (Å)	0.00	0.20	0.23
Binding energy (kcal/mol)	+192.91	+13.08	+14.07

**Table 5:** Characteristics of genital warts in the studied patients.

	Variables	Patient Group, N (%); *n* = 40
Duration (year)	Mean ± SD	3 ± 1.5
Site	Face	5 (12.5%)
	Arm	13 (23.5%)
	Leg	7 (17.5%)
	Trunk	3 (7.5%)
	Arm and leg	7 (17.5%)
	Leg and face	3 (7.5%)
	Trunk and face	1 (2.5%)
	Trunk and arm	1 (2.5%)
Extent	Range	5–30
	Mean ± SD	13.87 ± 7.8
Koebner phenomena	Yes	10 (25%)
	No	30 (75%)
VASI score	Range	5–80
	Mean ± SD	28.25 ± 20.7
VIDA score	0	6 (7.2%)
	1	3 (3.6%)
	2	8 (9.6%)
	3	13 (15.7%)
	4	10 (12.0%)

Data are presented as frequency (N) and percentage (%) or mean ± standard deviation (SD). VASI: Vitiligo Area Scoring Index; VIDA: Vitiligo Disease Activity.

## Data Availability

The original contributions presented in this study are included in the article. Further inquiries can be directed to the corresponding authors.

## Supplementary Materials

The following supporting information can be downloaded at https://www.mdpi.com/article/10.3390/biom15071053/s1: Table S1: SwissADME Predicted Properties of Vitexin.

## Abbreviations

The following abbreviations are used in this manuscript:

ARE	Antioxidant Response Element
Cq	Quantification Cycle
CRL	Cullin-RING Ligase
CUL3	Cullin-3
CV	Coefficient of Variation
FOXP	Forkhead Box Protein P
GAPDH	Glyceraldehyde-3-Phosphate Dehydrogenase
IL-6	Interleukin-6
miRNA	MicroRNA
miR-146a	MicroRNA-146a
NF-κB	Nuclear Factor Kappa-Light-Chain-Enhancer of Activated B Cells
NRF2	Nuclear Factor Erythroid 2-Related Factor 2
PPI	Protein–Protein Interaction
qRT-PCR	Quantitative Real-Time Polymerase Chain Reaction
RNA	Ribonucleic Acid
RMSD	Root Mean Square Deviation
ROS	Reactive Oxygen Species
RT	Reverse Transcription
SD	Standard Deviation
SOPs	Standard operating procedures
TNF-α	Tumor Necrosis Factor-Alpha
VIDA	Vitiligo Disease Activity Index
VASI	Vitiligo Area Scoring Index

## References

[1] Hlača N., Žagar T., Kaštelan M., Brajac I., Prpić-Massari L. (2022). Current Concepts of Vitiligo Immunopathogenesis. Biomedicines.

[2] Chang W.-L., Ko C.-H. (2023). The Role of Oxidative Stress in Vitiligo: An Update on Its Pathogenesis and Therapeutic Implications. Cells.

[3] Wang Y., Li S., Li C. (2019). Perspectives of New Advances in the Pathogenesis of Vitiligo: From Oxidative Stress to Autoimmunity. Med. Sci. Monit..

[4] Xu X., Jiang M., Zhang C.-F., Qiao Z., Liu W., Le Y., Wu J., Ma W., Xiang L. (2021). New insights into segmental vitiligo: A clinical and immunological comparison with nonsegmental vitiligo. Pigment. Cell Melanoma Res..

[5] AL-smadi K., Imran M., Leite-Silva V.R., Mohammed Y. (2023). Vitiligo: A Review of Aetiology, Pathogenesis, Treatment, and Psychosocial Impact. Cosmetics.

[6] Upadhya S., Andrade M.J., Shukla V., Rao R., Satyamoorthy K. (2025). Genetic and immune dysregulation in vitiligo: Insights into autoimmune mechanisms and disease pathogenesis. Autoimmun. Rev..

[7] Chen J., Li S., Li C. (2021). Mechanisms of melanocyte death in vitiligo. Med. Res. Rev..

[8] Ngo V., Duennwald M.L. (2022). Nrf2 and Oxidative Stress: A General Overview of Mechanisms and Implications in Human Disease. Antioxidants.

[9] Bellezza I., Giambanco I., Minelli A., Donato R. (2018). Nrf2-Keap1 signaling in oxidative and reductive stress. Biochim. Biophys. Acta (BBA)-Mol. Cell Res..

[10] Baird L., Yamamoto M. (2020). The Molecular Mechanisms Regulating the KEAP1-NRF2 Pathway. Mol. Cell. Biol..

[11] Cuadrado A., Rojo A.I., Wells G., Hayes J.D., Cousin S.P., Rumsey W.L., Attucks O.C., Franklin S., Levonen A.-L., Kensler T.W. (2019). Therapeutic targeting of the NRF2 and KEAP1 partnership in chronic diseases. Nat. Rev. Drug Discov..

[12] Cai W., Yang H. (2016). The structure and regulation of Cullin 2 based E3 ubiquitin ligases and their biological functions. Cell Div..

[13] Li X., Yang K.B., Chen W., Mai J., Wu X.Q., Sun T., Wu R.Y., Jiao L., Li D.D., Ji J. (2021). CUL3 (cullin 3)-mediated ubiquitination and degradation of BECN1 (beclin 1) inhibit autophagy and promote tumor progression. Autophagy.

[14] Lu L., He H., Feng J., Hu Z., Zhang S., Yang L., Liu Y., Wang T. (2024). Post-translational modification in the pathogenesis of vitiligo. Immunol. Res..

[15] Paganelli A., Cristofoletti C., Moro F., Corrente A., Colonna L., Scala E., Picardo M. (2025). Comprehensive Overview of Cytokine Interplay in Vitiligo: A Decade of Meta-Analyses Systematically Reviewed. Life.

[16] Lyu C., Sun Y. (2022). Immunometabolism in the pathogenesis of vitiligo. Front. Immunol.

[17] Saha S., Buttari B., Panieri E., Profumo E., Saso L. (2020). An Overview of Nrf2 Signaling Pathway and Its Role in Inflammation. Molecules.

[18] Lin X., Meng X., Song Z., Lin J. (2020). Nuclear factor erythroid 2-related factor 2 (Nrf2) as a potential therapeutic target for vitiligo. Arch. Biochem. Biophys..

[19] Boniface K., Seneschal J., Picardo M., Taïeb A. (2018). Vitiligo: Focus on Clinical Aspects, Immunopathogenesis, and Therapy. Clin. Rev. Allergy Immunol..

[20] Hushcha Y., Blo I., Oton-Gonzalez L., Mauro G.D., Martini F., Tognon M., Mattei M.D. (2021). microRNAs in the Regulation of Melanogenesis. Int. J. Mol. Sci..

[21] Saba R., Sorensen D.L., Booth S.A. (2014). MicroRNA-146a: A Dominant, Negative Regulator of the Innate Immune Response. Front. Immunol..

[22] Ruksha T.G., Komina A.V., Palkina N.V. (2017). MicroRNA in skin diseases. Eur. J. Dermatol. EJD.

[23] Dwivedi M., Kemp E.H., Laddha N.C., Mansuri M.S., Weetman A.P., Begum R. (2015). Regulatory T cells in vitiligo: Implications for pathogenesis and therapeutics. Autoimmun. Rev..

[24] Lu L.F., Boldin M.P., Chaudhry A., Lin L.L., Taganov K.D., Hanada T., Yoshimura A., Baltimore D., Rudensky A.Y. (2010). Function of miR-146a in controlling Treg cell-mediated regulation of Th1 responses. Cell.

[25] Babaei F., Moafizad A., Darvishvand Z., Mirzababaei M., Hosseinzadeh H., Nassiri-Asl M. (2020). Review of the effects of vitexin in oxidative stress-related diseases. Food Sci. Nutr..

[26] Li X.-S., Tang X.-Y., Su W., Li X. (2020). Vitexin protects melanocytes from oxidative stress via activating MAPK-Nrf2/ARE pathway. Immunopharmacol. Immunotoxicol..

[27] Jian Z., Li K., Song P., Zhu G., Zhu L., Cui T., Liu B., Tang L., Wang X., Wang G. (2014). Impaired activation of the Nrf2-ARE signaling pathway undermines H_2_O_2_-induced oxidative stress response: A possible mechanism for melanocyte degeneration in vitiligo. J. Investig. Dermatol..

[28] Hamzavi I., Jain H., McLean D., Shapiro J., Zeng H., Lui H. (2004). Parametric modeling of narrowband UV-B phototherapy for vitiligo using a novel quantitative tool: The Vitiligo Area Scoring Index. Arch. Dermatol..

[29] Feily A. (2014). Vitiligo Extent Tensity Index (VETI) score: A new definition, assessment and treatment evaluation criteria in vitiligo. Dermatol. Pract. Concept..

[30] Weiss M.M. (2020). Vitiligo: To biopsy or not to biopsy. Cutis.

[31] Du X., Li S., Yang K., Cao Y. (2023). Downregulation of Sonic hedgehog signaling induces G2-arrest in genital warts. Ski. Res. Technol..

[32] Bustin S.A., Benes V., Garson J.A., Hellemans J., Huggett J., Kubista M., Mueller R., Nolan T., Pfaffl M.W., Shipley G.L. (2009). The MIQE guidelines: Minimum information for publication of quantitative real-time PCR experiments. Clin. Chem..

[33] Livak K.J., Schmittgen T.D. (2001). Analysis of relative gene expression data using real-time quantitative PCR and the 2(-Delta Delta C(T)) Method. Methods.

[34] Maher S.A., Ismail N.A., Toraih E.A., Habib A.H., Gouda N.S., Gomaa A.H.A., Fawzy M.S., Helal G.M. (2022). Hair Follicle-Related MicroRNA-34a Serum Expression and rs2666433A/G Variant in Patients with Alopecia: A Cross-Sectional Analysis. Biomolecules.

[35] Fawzy M.S., Ibrahiem A.T., Bayomy N.A., Makhdoom A.K., Alanazi K.S., Alanazi A.M., Mukhlef A.M., Toraih E.A. (2023). MicroRNA-155 and Disease-Related Immunohistochemical Parameters in Cutaneous Melanoma. Diagnostics.

[36] Allen C.A., Payne S.L., Harville M., Cohen N., Russell K.E. (2007). Validation of quantitative polymerase chain reaction assays for measuring cytokine expression in equine macrophages. J. Immunol. Methods.

[37] Chesher D. (2008). Evaluating assay precision. Clin. Biochemist. Rev..

[38] Tiezzi F., Parker-Gaddis K., Cole J., Clay J., Maltecca C.J.P.O. (2015). Pathways identified by the Database for Annotation, Visualization and Integrated Discovery (DAVID version 6.7) in the Kyoto Encyclopedia of Genes and Genomes (KEGG). Dataset.

[39] Zhou G., Soufan O., Ewald J., Hancock R.E.W., Basu N., Xia J. (2019). NetworkAnalyst 3.0: A visual analytics platform for comprehensive gene expression profiling and meta-analysis. Nucleic Acids Res..

[40] Zogopoulos V.L., Saxami G., Malatras A., Papadopoulos K., Tsotra I., Iconomidou V.A., Michalopoulos I. (2022). Approaches in Gene Coexpression Analysis in Eukaryotes. Biology.

[41] Pettersen E.F., Goddard T.D., Huang C.C., Couch G.S., Greenblatt D.M., Meng E.C., Ferrin T.E. (2004). UCSF Chimera—A visualization system for exploratory research and analysis. J. Comput. Chem..

[42] Meng X.Y., Zhang H.X., Mezei M., Cui M. (2011). Molecular docking: A powerful approach for structure-based drug discovery. Curr. Comput.-Aided Drug Des..

[43] Daina A., Michielin O., Zoete V. (2017). SwissADME: A free web tool to evaluate pharmacokinetics, drug-likeness and medicinal chemistry friendliness of small molecules. Sci. Rep..

[44] Periasamy N., Ramesh V., Matlani M. (2023). A Case-Control Study of Serum and Tissue Catalase among Morphological Variants of Vitiligo. Indian J. Dermatol..

[45] Ocampo-Candiani J., Salinas-Santander M., Trevino V., Ortiz-López R., Ocampo-Garza J., Sanchez-Dominguez C.N. (2018). Evaluation of skin expression profiles of patients with vitiligo treated with narrow-band UVB therapy by targeted RNA-seq. An. Bras. Dermatol..

[46] Tuccinardi T., Poli G., Romboli V., Giordano A., Martinelli A. (2014). Extensive Consensus Docking Evaluation for Ligand Pose Prediction and Virtual Screening Studies. J. Chem. Inf. Model..

[47] Qiu L., Song Z., Setaluri V. (2014). Oxidative stress and vitiligo: The Nrf2-ARE signaling connection. J. Investig. Dermatol..

[48] Arowojolu O.A., Orlow S.J., Elbuluk N., Manga P. (2017). The nuclear factor (erythroid-derived 2)-like 2 (NRF2) antioxidant response promotes melanocyte viability and reduces toxicity of the vitiligo-inducing phenol monobenzone. Exp. Dermatol..

[49] Romano-Lozano V., Cruz-Avelar A., Peralta-Pedrero M.L. (2022). Nuclear Factor Erythroid 2-Related Factor 2 in Vitiligo. Actas Dermo-Sifiliográficas.

[50] Sorour N.E., Abd El-Kareem H.M., Ibrahim A.E., Salem R.M. (2021). Nuclear Factor Erythroid-2-related Factor 2 Gene Polymorphisms in Vitiligo. J. Clin. Aesthetic Dermatol..

[51] Natarajan V.T., Singh A., Kumar A.A., Sharma P., Kar H.K., Marrot L., Meunier J.-R., Natarajan K., Rani R., Gokhale R.S. (2010). Transcriptional Upregulation of Nrf2-Dependent Phase II Detoxification Genes in the Involved Epidermis of Vitiligo Vulgaris. J. Investig. Dermatol..

[52] Hassan A.M., Neinaa Y.M.E.-H., El-Bendary A.S., Zakaria S.S. (2019). MicroRNA-146a and Forkhead box protein 3 expressions in nonsegmental vitiligo: An insight into disease pathogenesis. J. Egypt. Women’s Dermatol. Soc..

[53] Campione E., Lanna C., Diluvio L., Cannizzaro M.V., Grelli S., Galluzzo M., Talamonti M., Annicchiarico-Petruzzelli M., Mancini M., Melino G. (2020). Skin immunity and its dysregulation in atopic dermatitis, hidradenitis suppurativa and vitiligo. Cell Cycle.

[54] Xie Y., Chu A., Feng Y., Chen L., Shao Y., Luo Q., Deng X., Wu M., Shi X., Chen Y. (2018). MicroRNA-146a: A Comprehensive Indicator of Inflammation and Oxidative Stress Status Induced in the Brain of Chronic T2DM Rats. Front. Pharmacol..

[55] Shi Y.L., Weiland M., Li J., Hamzavi I., Henderson M., Huggins R.H., Mahmoud B.H., Agbai O., Mi X., Dong Z. (2013). MicroRNA expression profiling identifies potential serum biomarkers for non-segmental vitiligo. Pigment. Cell Melanoma Res..

[56] Wang Y., Wang K., Liang J., Yang H., Dang N., Yang X., Kong Y. (2015). Differential expression analysis of miRNA in peripheral blood mononuclear cells of patients with non-segmental vitiligo. J. Dermatol..

[57] Shahmatova L., Tankov S., Prans E., Aab A., Hermann H., Reemann P., Pihlap M., Karelson M., Abram K., Kisand K. (2016). MicroRNA-155 is Dysregulated in the Skin of Patients with Vitiligo and Inhibits Melanogenesis-associated Genes in Melanocytes and Keratinocytes. Acta Derm.-Venereol..

[58] Nakasa T., Miyaki S., Okubo A., Hashimoto M., Nishida K., Ochi M., Asahara H. (2008). Expression of microRNA-146 in rheumatoid arthritis synovial tissue. Arthritis Rheum..

[59] Tembhre M.K., Parihar A., Sharma V., Sharma A., Chattopadhyay P., Gupta S. (2014). Alteration in regulatory T cells and programmed death (PD)1 expressing regulatory T cells in active generalized vitiligo and their clinical correlation. Br. J. Dermatol..

[60] Kidir M., Karabulut A.A., Ercin M.E., Atasoy P. (2017). Regulatory T-cell cytokines in patients with nonsegmental vitiligo. Int. J. Dermatol..

[61] Ben Ahmed M., Zaraa I., Rekik R., Elbeldi-Ferchiou A., Kourda N., Belhadj Hmida N., Abdeladhim M., Karoui O., Ben Osman A., Mokni M. (2012). Functional defects of peripheral regulatory T lymphocytes in patients with progressive vitiligo. Pigment. Cell Melanoma Res..

[62] Mitra S., De Sarkar S., Pradhan A., Pati A.K., Pradhan R., Mondal D., Sen S., Ghosh A., Chatterjee S., Chatterjee M. (2017). Levels of oxidative damage and proinflammatory cytokines are enhanced in patients with active vitiligo. Free. Radic. Res..

[63] Singh S., Singh U., Pandey S.S. (2012). Serum concentration of IL-6, IL-2, TNF-α, and IFNγ in Vitiligo patients. Indian J. Dermatol..

[64] Sushama S., Dixit N., Gautam R.K., Arora P., Khurana A., Anubhuti A. (2019). Cytokine profile (IL-2, IL-6, IL-17, IL-22, and TNF-α) in vitiligo—New insight into pathogenesis of disease. J. Cosmet Dermatol..

[65] Yang X., Yan L., Ha D., Qu L., Liu L., Tao Y. (2019). Changes in sICAM-1 and GM-CSF levels in skin tissue fluid and expression of IL-6, IL-17 and TNF-α in blood of patients with vitiligo. Exp. Ther. Med..

[66] Moretti S., Spallanzani A., Amato L., Hautmann G., Gallerani I., Fabiani M., Fabbri P. (2002). New Insights into the Pathogenesis of Vitiligo: Imbalance of Epidermal Cytokines at Sites of Lesions. Pigment Cell Res..

[67] Lu Y., Yu T., Liu J., Gu L. (2018). Vitexin attenuates lipopolysaccharide-induced acute lung injury by controlling the Nrf2 pathway. PLoS ONE.

[68] Song J., Wang H., Sheng J., Zhang W., Lei J., Gan W., Cai F., Yang Y. (2023). Vitexin attenuates chronic kidney disease by inhibiting renal tubular epithelial cell ferroptosis via NRF2 activation. Mol. Med..

[69] Huang X., Li T., Li S. (2023). Encapsulation of vitexin-rhamnoside based on zein/pectin nanoparticles improved its stability and bioavailability. Curr. Res. Food Sci..

[70] Gu C., Liu Z., Yuan X., Li W., Zu Y., Fu Y. (2017). Preparation of Vitexin Nanoparticles by Combining the Antisolvent Precipitation and High Pressure Homogenization Approaches Followed by Lyophilization for Dissolution Rate Enhancement. Molecules.

[71] Eleftheriadou V., Delattre C., Chetty-Mhlanga S., Lee C., Girardat-Rotar L., Khan I., Mathew A., Thompson A.R. (2024). Burden of disease and treatment patterns in patients with vitiligo: Findings from a national longitudinal retrospective study in the UK. Br. J. Dermatol..

[72] Clemente Hernández B., Muelas Rives I., Gracia Cazaña T., Álvarez Salafranca M., Poblador-Plou B., Laguna-Berna C., Moreno Juste A., Gimeno-Miguel A., Gilaberte Y. (2025). Comorbidities Associated with Vitiligo: Results from the EpiChron Cohort. J. Clin. Med..

