# Supplementary material for: Cullin-3 and Regulatory Biomolecules Profiling in Vitiligo: Integrated Docking, Clinical, and In Silico Insights
Source: Biomolecules. 2025 Jul 21;15(7):1053. doi: 10.3390/biom15071053 (PMC12294106; doi:10.3390/biom15071053)
Supplement: Supplementary file 1 [file biomolecules-15-01053-s001.zip › biomolecules-3720975-supplementary.pdf]

Cullin-3 and Regulatory Biomolecules Profiling in Vitiligo: Integrated Docking, Clinical, and In Silico Insights

TableS1. SwissADME Predicted Properties of Vitexin

Category	Property	Vitexin Value / Prediction
Physicochemical	Molecular weight	432.38 g/moL
	TPSA	181.05 Å^2
	Hydrogen bond donors	7
	Hydrogen bond acceptors	10
	LogP (consensus)	– 0.02
Pharmacokinetics	GI absorption	Low
	BBB permeation	No
	P-gp substrate	No
	CYP inhibition	None predicted
	Water solubility (Log S, ESOL)	– 2.38
Drug-likeness	Lipinski rule	✕ (1 violation: H-bond donors)
	Veber rule	✕ (TPSA > 140 Å^2)
	Egan rule	✕
	Muegge rule	✕
Safety filters	PAINS alerts	✕ None
	Brenk alerts	✕ None
	Lead-likeness	✕
	Bioavailability score	0.17

(X): Indicates a rule violation or negative prediction; TPSA: Topological Polar Surface Area; GI: Gastrointestinal; BBB: Blood–Brain Barrier; P-gp: P-glycoprotein; CYP: Cytochrome P450 enzymes; PAINS: Pan Assay Interference Compounds; LogP: Octanol-water partition coefficient (lipophilicity).
